# “An unprecedented occurrence: a case report of pulmonary hypertension manifestation in Donohue syndrome”

**DOI:** 10.1186/s12887-024-04714-1

**Published:** 2024-05-11

**Authors:** Ahmed Shamil Hashim, Mustafa Najah Al-Obaidi, Ahmed Dheyaa Al-Obaidi, Saleh Abdulkareem Saleh, Hashim Talib Hashim, Mina Al Saeedi, Basma Ataallah

**Affiliations:** 1 College of Medicine, University of Baghdad, Baghdad, Bab Al-Muaddam Iraq; 2 College of Medicine, University of Warith Al-Anbiyaa, Karbala, Iraq; 3 College of Medicine, University of Al-Mustansiriyah, Baghdad, Iraq; 4 Methodist Willbrook Hospital, Houston, USA

**Keywords:** Donohue syndrome, Leprechaunism, Hyperinsulinism, Insulin receptor, Insulin resistance, Pulmonary hypertension

## Abstract

**Introduction:**

Donohue syndrome (DS), also referred to as leprechaunism, is a remarkably uncommon autosomal recessive disorder that primarily affects the endocrine system. Its incidence rate is exceedingly low, with only 1 case reported per 4 million live births. The syndrome is distinguished by a series of characteristic clinical features.

**Case presentation:**

We present a case of a twenty-month-old male with DS who experienced a range of dysmorphic and clinical features with the involvement of multiple systems. These features include skin hyperpigmentation, hypertrichosis, distinct facial features, abdominal distension, and microcephaly, with the involvement of the endocrine, renal, respiratory, and cardiac systems.

**Conclusion:**

The primary features of DS involve severe insulin resistance and growth abnormalities, the association with pulmonary hypertension (PHTN) has not been reported before. This finding adds more complexity to the condition. To the best of the author’s knowledge, this is the first report for a patient with DS who has PHTN. Further investigation is required since the mechanisms behind the development of PHTN in DS are not entirely understood. Shedding light on this association will contribute to better management strategies and outcomes for affected patients.

## Introduction

Donohue syndrome (DS), also known as leprechaunism, is an extremely rare disorder that affects the endocrine system [1]. It is caused by a mutation in the Insulin Receptor gene located on chromosome 19p13, and is inherited in an autosomal recessive pattern. DS is a rare medical condition that has a low incidence rate of 1 in every 4 million live births [2]. Donohue and Uchida first described the DS in 1948 and 1954, respectively. DS is characterized by a number of distinguishing clinical features, including a depressed nasal bridge, thick lips, low-set large ears, and hypertelorism of the orbits [3]. There have only been a few documented cases of DS in the medical literature; furthermore, none of them report an association with pulmonary hypertension (PHTN). In this case report, we present a case of DS in a twenty-month-old male who found out to have PHTN during assessment; moreover, the child exhibited a spectrum of dysmorphic and clinical features with the involvement of multiple systems.

## Case presentation

**Fig. 1:**
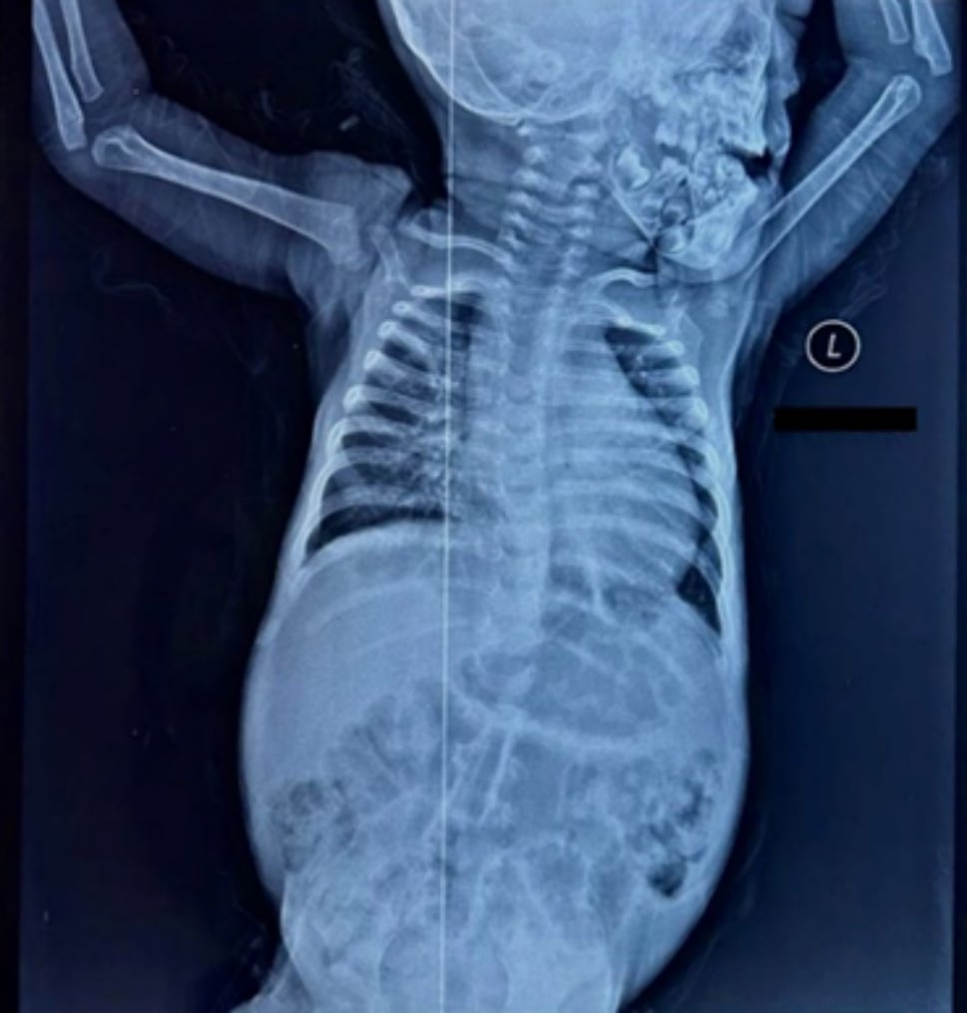
chest x-ray showed bilateral diffuse patchy infiltrates (white arrows) and cardiomegaly (black arrows showing cardiac silhouette transverse diameter more than 0.5 of the chest transverse diameter)

A three-months old male was brought to our hospital by his parents due to frequent bowel motion and vomiting lasting for one month. At that time, the patient had lost 1.5 kg of his weight, had an elevated axillary corrected body temperature of 38.3 C, and exhibited skin hyperpigmentation in various regions, including the axilla, neck, peri-umbilical area, chest, and abdomen. The patient also had hypertrichosis on the face and body and distinct facial features, including bulging eyes, thick lips, large, low-set ears, and upturned nostrils. Abdominal distension, microcephaly, and a short pedal length were also noted during the physical examination. (as shown in Fig. 1A and B).

The patient was a product of consanguineous marriage, and at delivery he weighed 3100 g. He also had a deceased male sibling with similar facial features and excessive hair growth on the head and body who passed away at 4 months of age. The other siblings (three sisters and one brother) were all healthy. During the mother’s 38-week pregnancy, there were no complications apart from a resolved COVID-19 infection during the second trimester. The delivery was also normal and without any complications. Due to the family history and dysmorphic features, laboratory investigations were performed at delivery, revealing a blood sugar of 191.2 mg/dL (less than 150 mg/dL), cortisol level of 85.96 µg/dL (1.6 to 9.1 µg/dL), ACTH level of 30.6 pg/mL (5–46 pg/mL), 17-OH progesterone level of 7.8 ng/mL (less than 1 ng/mL), DHEAS level of 342 µg/dL (10–500 µg/dL), TSH level of 1.2 µIU/mL (0.6 to 7.3 µIU/mL), thyroxine (T4) level of 0.09 µg/dL (7.2 to 15.7 µg/dL), and hyperinsulinemia. The subsequent days revealed a fasting blood sugar of 41 mg/dL and postprandial blood glucose value of 212 mg/dL. The abdominal ultrasonography revealed mild to moderate bilateral renal echogenicity associated with crystals in the calyceal system. A pediatric nephrologist was consulted, and the patient was sent for investigations, which revealed a pH of 7.41 (7.35–7.45) and a bicarbonate level of 17.6 mmol/L (18–24 mmol/L). Renal function tests were within the reference range. An echocardiogram was also done, which revealed a patent foramen ovale. Based on the patient’s history, physical examination findings, and elevated blood glucose levels, DS was suspected, and the patient was started on hydrocortisone and insulin therapy. However, due to the limited resources in the country, genetic testing couldn’t be performed. The patient was scheduled for regular follow-up appointments at the hospital’s outpatient clinic. At 12-months of age, another echocardiogram was performed, which revealed mild left ventricular hypertrophy.

**Fig. 2:**
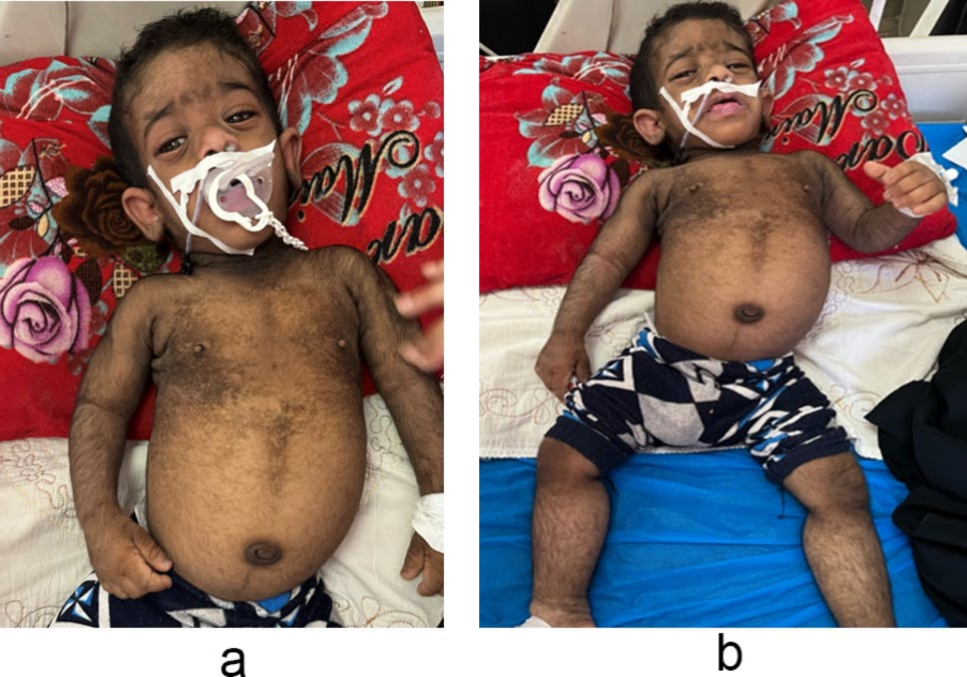
(**A** and **B**): showcases the patient’s clinical features, including skin hyperpigmentation at the axilla, neck, peri-umbilical area, chest, and abdomen, hypertrichosis on the face and body, distinct facial features including bulging eyes, thick lips, large low-set ears, and upturned nostrils, as well as abdominal distension, and microcephaly

At the time of writing this study, the patient was 20-months old and he was brought to the hospital due to shortness of breath for two-days duration. He also had occasional cyanosis of the lips, coughing, fever, abnormal breathing sounds, and reduced urine output. The patient appeared distressed, irritable, and dyspneic. On physical examination, the vital signs revealed a respiratory rate of 44 breaths per minute, an oxygen saturation of 89%, a heart rate of 147 beats per minute, and an axillary corrected body temperature of 38.7 °C. Chest auscultation revealed bilateral diffuse crepitations and rhonchi, while the chest x-ray showed bilateral diffuse patchy infiltrates and cardiomegaly *(as shown in* Fig. 2*)*. The patient’s anthropometric measurements were obtained, revealing a length of 72 cm and a weight of 9000 g. A blood investigation was also requested and showed an elevated white blood cell count of 19,200/mm^3^(6000/mm^3^–17,500/mm^3^) and an elevated C-reactive protein of 48 mg/dL (less than 10 mg/dL). According to the previous findings, the patient was diagnosed with interstitial pneumonia and treated with oxygen therapy, IV fluids, and intravenous vancomycin and meropenem.

**Fig. 3:**
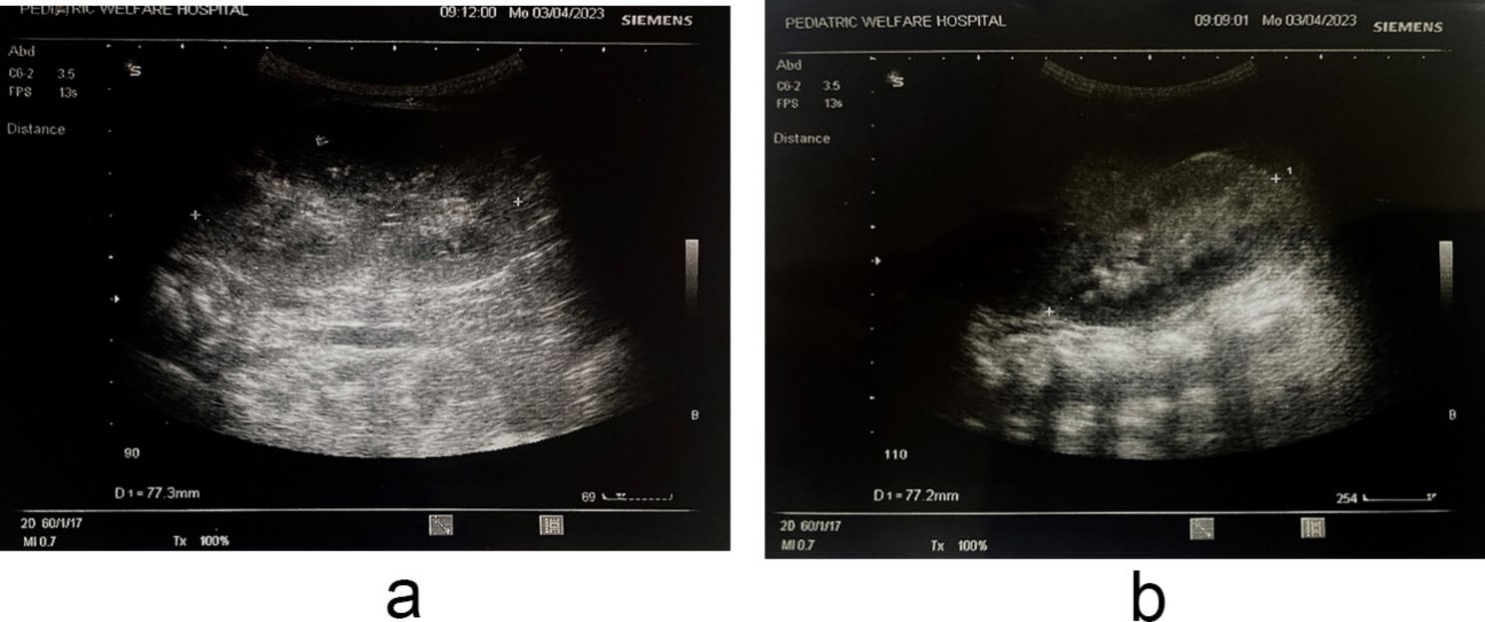
(**A**) An abdominal ultrasonography demonstrates mild ascites. (**B**) there are also multiple echogenic foci seen in the renal pyramid, suggesting grade I medullary nephrocalcinosis

Following the patient’s most recent presentation for shortness of breath, the renal function tests showed a blood urea level of 66 mg/dL (7–20 mg/dL) and a creatinine level of 0.18 mg/dL (0.4–1.2 mg/dL), while the random blood sugar was 91 mg/dL (less than 200 mg/dL), and Blood gas analysis revealed a corrected pH of 7.32 (7.35–7.45), pCO2 of 78.6 mmHg (35–45 mmHg), pO2 of 24.7 mmHg (75–100 mmHg), HCO3 of 35.1 mmol/L (22–28 mmol/L), and lactate of 3.3 mmol/L (0.5–2.2 mmol/L). An abdominal ultrasonography demonstrates mild ascites and enlargement of both kidneys measuring 7.7 cm with an increase in parenchymal echogenicity (grade III) and there is no corticomedullary dissociation. There are also multiple echogenic foci seen in the renal pyramid, suggesting grade I medullary nephrocalcinosis (as shown in Fig. 3A and B). The echocardiography revealed mild left ventricular hypertrophy, right-sided dilation, severe tricuspid regurgitation (PG = 64 mmHg), and severe PHTN. The patient was started on sildenafil, gradually improved after the initiation of antibiotics, and was discharged after six days with scheduled follow-up in the outpatient clinic.

## Discussion

DS or leprechaunism is an exceedingly rare genetic disease that is passed down through an autosomal recessive inheritance pattern. It occurs in less than one per million cases. It is characterized by insulin receptor gene mutations that cause disabled or non-functional insulin receptors, leading to complete resistance to insulin [4].

Children who have DS exhibit peculiar features that include a combination of physical characteristics and metabolic symptoms. It involves but is not limited to intrauterine and postnatal growth retardation, hypertrichosis, acanthosis nigricans, lipoatrophy, clitoromegaly, virilization, emaciation, abdominal distension, microcephaly, fasting hypoglycemia, postprandial hyperglycemia, insulin resistance, and hyperinsulinemia [5].

Our patient in this case report exhibits the classical characteristics of DS including postprandial hyperglycemia, abdominal distension, hirsutism, acanthosis nigricans, and craniofacial abnormalities represented by microcephaly, bulging eyes, thick lips, large, low-set ears, and upturned nostrils.

As a consequence of extreme insulin resistance and high circulating insulin levels, patients usually develop functional and structural defects in many body organs. For example, heart involvement can lead to hypertrophic cardiomyopathy and/or congenital heart defects such as ventricular septal defects, therefore, screening with echocardiography should be done for every child who has DS [5]. The explanation behind the development of hypertrophic cardiomyopathy in those patients is not yet determined, but it is thought to be due to high insulin concentrations that act through the insulin-like growth factor-I (IGF-1) receptor, which is also present in the heart [6, 7]. However, an important complication that occurs in DS that has not been reported before is PHTN. In this child, after requesting echocardiography, it revealed mild left ventricular hypertrophy, right-sided dilation, severe tricuspid regurgitation, and severe PHTN.

PHTN is characterized by sustained increased blood pressure in the pulmonary arteries (> 20 mmHg as determined by right heart catheterization), which can cause a higher level of resistance and strain on the right side of the heart. It can result from various underlying factors, including genetic disorders, congenital heart diseases, and pulmonary vascular abnormalities [8]. In the case of DS, the exact mechanisms involved and the prevalence of PHTN are not well understood, given the rarity of the syndrome. DS can be associated with many congenital heart diseases that can lead to the possibility of developing PHTN. In our case, the patient was regularly followed up by echocardiography and screening for heart abnormalities, revealing only patent foramen ovale and mild left ventricular hypertrophy, which are both unlikely to lead to PHTN. Furthermore, Insulin resistance and dysregulation of growth signaling factors, such as IGF-1 receptors, could potentially affect pulmonary vascular remodeling and function, making them more susceptible to developing PHTN [9].

In DS, the PHTN can further exacerbate the already compromised cardiovascular system, leading to increased strain on the heart and the potential for right-sided heart failure. Additionally, PHTN can worsen respiratory symptoms and oxygenation, potentially impacting the overall prognosis and management of the syndrome. This was evident in our case, where a child with interstitial pneumonia was experiencing difficulty breathing and was at risk of respiratory compromise.

Management of DS carries significant challenges due to its complex pathophysiology. The rationale for using hydrocortisone with insulin in the management of DS lies in their complementary mechanisms of action. While insulin directly addresses hyperglycemia by enhancing glucose uptake and preventing hepatic glucose production, hydrocortisone aims for increasing sensitivity to insulin by enhancing insulin receptor signaling, promoting glucose uptake in peripheral tissues, and suppressing hepatic glucose production, too. Additionally, glucocorticoids can suppress the inflammation associated with insulin resistance, thereby potentially mitigating the systemic consequences of DS [10]. Therefore, by concurrently addressing both hyperglycemia and insulin resistance, combination therapy with hydrocortisone and insulin may offer synergistic effects in improving glycemic control and ameliorating the metabolic derangements associated with DS [11]. In this patient, hydrocortisone was also prescribed due to the high level of 17-OH progesterone, which raised concerns regarding potential issues related to hyperandrogenism.

Given the rarity of both DS and its association with PHTN, there is limited evidence concerning optimal management strategies. However, in our case, the patient was treated with sildenafil and the appropriate antibiotics for the pneumonia and then scheduled for further follow-up evaluation.

## Conclusion

The primary features of DS involve severe insulin resistance and growth abnormalities, the association with PHTN has not been reported before. This finding adds more complexity to the condition. To the best of the author’s knowledge, this is the first report for a patient with DS who has PHTN. Further investigation is required since the mechanisms behind the development of PHTN in DS are not entirely understood. Shedding light on this association will contribute to better management strategies and outcomes for affected patients.

## Data Availability

The datasets used and/or analysed during the current study are available from the corresponding author on reasonable request.
